# A comparison between a patient-specific bone regenerative implant and the osteochondral allograft procedure in a Hill-Sachs lesion, a cadaveric study

**DOI:** 10.1016/j.xrrt.2025.100591

**Published:** 2025-10-07

**Authors:** Michał S. Gałek-Aldridge, Koen Willemsen, Sophie H. Nelissen, Bart C.H. van der Wal, Jos Malda, Michel P.J. van den Bekerom, Arthur van Noort

**Affiliations:** a Department of Orthopaedics, University Medical Center Utrecht, Utrecht, The Netherlands; b Faculty of Veterinary Medicine, Department of Clinical Sciences, Utrecht University, Utrecht, The Netherlands; c Regenerative Medicine Center Utrecht, Utrecht, The Netherlands; d Shoulder and Elbow Unit, Joint Research, Department of Orthopaedic Surgery, OLVG, Amsterdam, The Netherlands; e Faculty of Behavioral and Movement Sciences, Department of Human Movement Sciences, Vrije Universiteit, Amsterdam, The Netherlands; f Department of Orthopaedic Surgery, Spaarne Gasthuis, Haarlem, The Netherlands; g Department of Orthopaedics and Sports Medicine, Erasmus University Medical Center, Rotterdam, The Netherlands

**Keywords:** 3D-printed implant, Bone regeneration, Hill-Sachs lesion, Humeral head reconstruction, Shoulder instability, Osteochondral allograft, Biocompatible scaffold and joint preservation

## Abstract

**Background:**

Anterior shoulder instability with >30% humeral bone loss is typically treated with an osteochondral allograft (OCA), though complications and reoperation rates remain high (20%-30%). New methods such as 3D printing are being researched to mitigate these results. This study compares the surface geometry and biomechanical integrity of a 3D-printed biodegradable, patient-specific bone regenerative implant (O3D) to traditional OCA in the treatment of Hill-Sachs lesions (HSLs).

**Methods:**

In 14 cadaveric shoulders, HSLs were created in a uniaxial biomechanical set-up and confirmed using imaging. The shoulders were randomized over 2 groups: group A, OCA surgery, and group B, magnesium phosphate-polycaprolactone 3D-printed implant (O3D). After the reconstruction of the HSLs, imaging was performed to measure surface morphology and articular congruence. Finally, uniaxial biomechanical testing was performed to measure postimplantation stability.

**Results:**

The average force needed to create a HSL was 1120 N. Implant surface area and joint surface area showed no significant difference between the groups (*P* = .69 and *P* = .48). Articular step-off and implantation gap showed no significant difference (*P* = .67 and *P* = .54). However, O3D demonstrated significantly better joint congruence (1.26 ± 0.29 mm) than OCA (3.17 ± 1.43 mm, *P* = .044). Breakout compression forces were not significantly different (*P* = .80) between the groups: OCA (152 ± 91 N) vs. O3D (144 ± 37 N). Micro computed tomography revealed differing failure mechanisms: cortical compression in OCA vs. layer deformation in O3D, reflecting their respective architectures.

**Conclusion:**

Both OCA and O3D implants effectively restored joint integrity in large HSLs. The O3D implant showed superior congruence and equivalent biomechanical performance, illustrating a 3D-personalized, regenerative alternative to allografts.

Anterior shoulder instability encompasses both dislocation, fear of dislocation, and subluxation events, predominantly affecting young, athletic individuals.^9^^,^^20^^,^^23^ In 42%-94% of anterior instability events, Hill-Sachs lesions (HSLs) occur, with up to 100% in those with recurrent instability.^18^^,^^20^ An HSL is defined as an osseous defect resulting from the impact of the glenoid rim on the posterolateral aspect of the articular surface of the humeral head (Fig. 1). This deformity alters shoulder joint anatomy, initially inducing shoulder pain with a possible loss of range of motion of the humeral joint. The proportion of HSLs increases with recurrent shoulder dislocations, resulting in a higher prevalence and greater clinical significance.^9^^,^^23^

**Figure 1:**
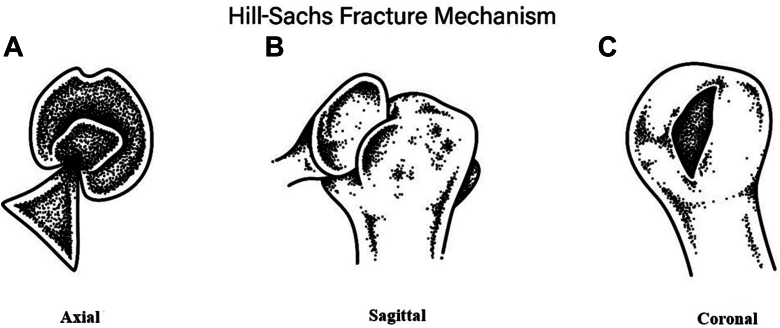
The Hill-Sachs fracture mechanism shown in (**A**, left) transverse view, with the pointed glenoid rim impinging on the humeral head; (**B**, middle) the anterior view of the trauma; and (**C**, right) the resulting bony impression fracture of the humeral head.

Following an initial dislocation (Fig. 1, *A* and *B*), the severity of HSLs, bony defects (Fig. 1, *C*), and associated ligament and labral abnormalities increases.^10^^,^^11^^,^^16^^,^^21^ Recurrent dislocation management focuses on regaining the maximum range of motion in the shoulder joint without subluxation. Treatment options range from conservative to surgical options. In surgical cases, the size and on-/off-track location of the HSL defect guide the treatment decisions.^12^ A larger HSL, *ie*, involvement of >40% of the articular surface, increases the likelihood of requiring surgical reconstruction with bone grafting because of compromised shoulder stability.^3^^,^^4^^,^^6^

An often-performed technique for reconstruction of a >40% articular surface HSLs is osteochondral allograft (OCA) transplantation.^3^^,^^5^ When OCA is successful, long-term follow-up shows acceptable functional results.^13^^,^^25^^,^^26^ However, postoperative complications for OCA—including avascular necrosis of the humeral head, collapse, persistent pain, stiffness and catching, and nonunion—are seen in 20% up to 74% of the cases.^13^^,^^25^^,^^26^ Concurrently postoperative arthropathy resulting from recurrent luxations has an incidence from 33% to 80%.^13^^,^^25^ This results in conversion to shoulder arthroplasty reported in up to 53% in 4.8 ± 4.7 years.^13^

To prevent partial avascular necrosis, allograft scarcity, nonunion, and graft resorption, 3D printing technologies have emerged as a promising alternative for OCA.^2^^,^^15^^,^^24^ 3D-printed implants enable patient-specific implants and have been linked to reducing operation times and infection risk.^2^^,^^15^ The drawback of these 3D-printed implants is the cost inefficiency of production and difficult to predict biodegradation potentially affecting long-term congruency. 3D printing techniques using magnesium phosphate and polycaprolactone (MgP-PCL 30%) ink have demonstrated in vivo osteoregenerative activity in an equine model.^8^ This cadaveric study explores the potential of such a biodegradable, regenerative bone implant produced by 3D printing to restore the original anatomy of the humeral head and thus restore stability. The potential drawbacks of 3D-printed implants are unpredictable biodegradation affecting long-term congruency.

The objectives of this study were to (1) compare the postprocedure morphology of this 3D printed implant with the morphology of the established OCA procedure and (2) to compare the biomechanical resilience of the 3D-printed biodegradable implant with the OCA procedure in a biomechanical setup.

## Study design

This study had repeated measurements of experimental design to adequately compare the surface geometry of the native humerus, the allograft augmented HSL, and the 3D-printed HSL. The following steps were undertaken in this study:

### Humeral head specimen collection

Nine fresh-frozen cadavers (4 women and 5 men) that were obtained using the university body donation program were used for our experiments. Shoulders with moderate to severe shoulder osteoarthritis or bone deformity were excluded by fluoroscopic examination. A total of 14 shoulders were used, with an average age of 82.5 years (range, 72-93 yr). They were disarticulated at the glenohumeral joint, avoiding damage to the humeral articular surface. All soft tissues, such as the glenohumeral joint capsule and glenoid labrum, were removed. The humeral head was osteotomized 1 cm distal from the beginning of the humeral neck. In between experiments, the humeri were stored in the freezer at −30°C.

### Specimen randomization

**Figure 2:**
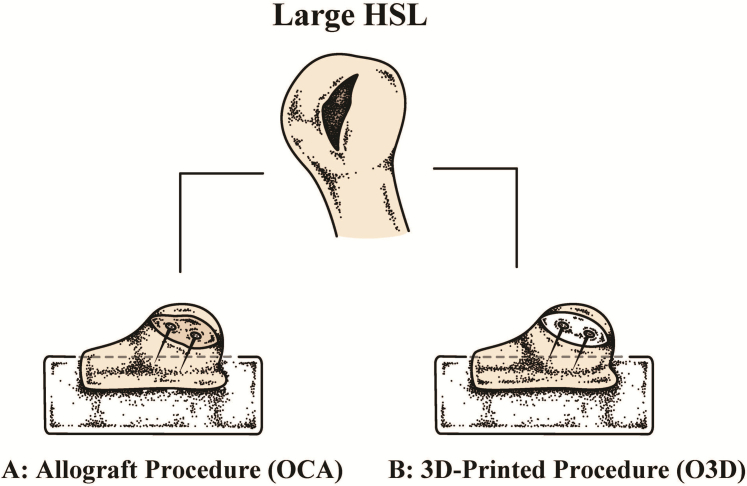
The experimental setup. After randomization, 2 groups were assigned: group A — allograft repair (OCA), resected from the femoral head; and group B — 3D-printed MgP-PCL implant repair (O3D), fabricated using the bone regenerative ceramic-polymer ink. Each group consisted of 7 shoulders. Both grafts were stabilized using 2 cannulated locking screws of equal length. *OCA*, osteochondral allograft; *HSL*, Hill-Sachs lesion; *O3D*, osteoregenerative 3D-printed implant.

The shoulders were randomized by age and sex to either group A: allograft repair (7 shoulders) or group B: MgP-PCL repair (7 shoulders) (Fig. 2).

### Specimen preparation

The shoulders were fixated in epoxy resin so that the articular surface next to the bare area (the posterior humeral head not covered by the articular cartilage) was pointing upwards, as described by Itoi et al.^12^

### Imaging and postprocessing

At the start, computed tomography (CT) scans (Brilliance 64 CT, Philips, Eindhoven, Netherlands) of all shoulders were made. All scans were made with a slice thickness of 0.9 mm (250 mAs, 120 kV). Data of the CT scans were analyzed by image processing software (Materialise Mimics Medical 21.0; Materialise, Leuven, Belgium). The software was used to make a 3D model of the native humerus and segmentate the predefect 3D model using a semiautomatic protocol utilizing standardized bone threshold values (≥226 Hounsfield units).

### Simulated large Hill-Sachs lesion creation

Specimens were thawed for 1 day before the creation of the Hill-Sachs defect and were kept moist with NaCl 0.9% solution during the tests. A LR5K Universal Testing Machine equipped with a XLC 5 kN load cell (Lloyd Instruments Inc., Hampshire, UK) was used to create the Hill-Sachs defect in a similar manner as described by Itoi et al.^12^ A custom-made triangular-shaped sawbone (representing a glenoid) was fixated in the biomechanical compression arm so that the sawbone, with a 90° impaction angle faced toward the bare area of the epoxy-fixated humeral head that was fixed in a vice. All shoulders underwent creation of compression fractures of at least until a depth of 15 mm was reached (large HSL^5^), 1 cm medial to the bare area.^12^ The Hill-Sachs compression tests were performed at a speed of 2.0 mm/s with a preload of 5 N. The peak force for each specimen was extracted.

### Imaging

After creating the HSL, a second CT scan of the shoulders was made with the same parameters as earlier described. Measurements of the length, width, and depth of the defect were done as described by Ozaki et al.^17^

## Group A: femoral head allograft harvest (osteochondral allograft)

Allograft preparation: seven fresh-frozen femoral heads were used from 4 cadaveric specimens, (average age 81 yr). The bone integrity was assessed by visual inspection and excluded if signs of osteoporosis or arthritis were present.

## Group B: magnesium phosphate -polycaprolactone 30% scaffold extrusion-based 3D printing

The HSL defect, as measured on imaging, was 3D reconstructed using 3D modelling software (Materialise 3 Matic Medical 13.0; Materialise, Leuven, Belgium) to design an HSL repair scaffold. Using computer-aided design Boolean subtraction, 2 perpendicular cuts were made to simulate an osteotomy in the humeral head, creating a virtual defect designed to accommodate the 3D-printed implant. The computer-aided design surface reconstruction tool was then used to compare the predefect and postdefect CT scans, allowing for the precise outline of the 3D implant to be generated. To ensure a secure press-fit into the actual humeral HSL defect, the virtual implant was subsequently enlarged by 1 mm on each side.^1^ The internal architecture of 0.7 mm interfiber spacing was used, corresponding to a 27.3% scaffold porosity. Perimeter placement was outside. The used angles were 0, 0, 90, and 90, as previously reported by Golafshan et al.^8^ These implants fabricated using a magnesium phosphate (70%)–PCL (30%) biomaterial ink through an extrusion-based method with the R200 3D Printer (RegenHu, Villaz Saint-Pierre, Switzerland).^7^^,^^8^ To understand in what direction the layers of the implants should be, 3D-printed a subtest was performed to analyze the optimal printing direction for the scaffolds. The printing orientation of the 3D-printed scaffold was further investigated for the 27.3% scaffold porosity implant. The scaffolds were printed in 2 orientations: group A: 45° toward the Z-direction, and group B: 0° toward the Z-direction. A 3D-printed nylon support that simulates a humeral head was used to hold the specimen in the right biomechanical orientation. The preload was 5 N, the rate 2 mm/min, and the tested displacement was 10 mm. Finally, a compression test was performed using the earlier mentioned Loyd universal testing machine.

### Surgical implantation

One day before the experiment, the specimens were thawed and were kept moist with NaCl solution during the tests, and were covered with wet towels when not used.

The surgical procedures were performed by a board-certified shoulder surgeon using a standard Shoulder Surgery Wet Set (Johnson and Johnson, Zug, Switzerland). This set included 6 K-wires of varying diameters (1.25 mm and 1.1 mm), a fine saw, a drill, and 36 cannulated Herbert screws (3.0 mm and 3.2 mm) without washers. Throughout the procedures, tissue hydration was maintained using 0.9% NaCl physiological solution.

In each fixed shoulder, a wedge cut was made with 2 perpendicular osteotomies to resect the edges of the Hill-Sachs defect and to prepare the humeral head for allograft or 3D scaffold implantation.

Group A: OCA. The allografts were manually reshaped to fit into the surgically prepared 90° wedge cut. The allograft was introduced, press-fit in place, and fixed using 2 self-tapping cannulated Herbert screws (3.0-3.2 mm).

Group B: 3D-printed MgP-PCL scaffold implants. Each 3D scaffold was introduced, press-fit in place, and fixed using 2 self-tapping cannulated Herbert screws (3.0-3.2 mm).

### Imaging, postprocessing, and measurements

**Figure 3:**
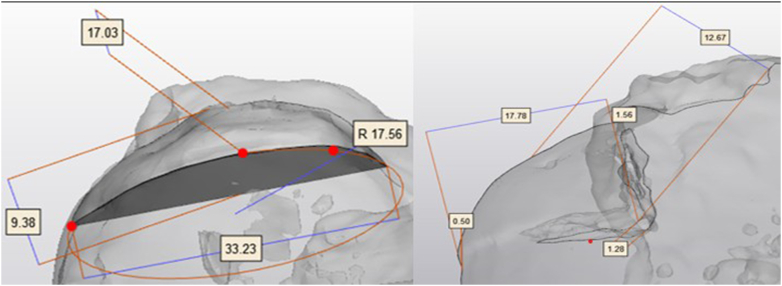
The imported postimplantation CT scan illustrates the measurement method. (**A**) (*left*): Measurement of the group B MgP-PCL implant height, width, radius, and length. (**B**) (*right*): Measurement of the group A allograft wedge, including articular step-off (0.5 mm), implantation gap (1.26 mm), and congruence step-off (1.56 mm). *CT*, computed tomography; *MgP-PCL*, magnesium phosphate polycaprolactone.

After the implantation procedure, a CT scan of the shoulders was made and another bone segmentation was performed. Next a stereolithography file format was uploaded into 3-Matic (Materialise B.V., Materialise, Leuven, Belgium). A perpendicular midline plane was generated in the middle of a line connecting the 2 outermost points of the allograft/scaffold. Using this midplane, a cut-through section of the humeral head and allograft/scaffold was made. On this cut-through section, measurements of the surface area, joint surface area, congruence, as well as length, radius, width, and depth of the allograft and scaffold were performed as described by Ozaki et al^17^ (Fig. 3, *A*).

The articular step-off is measured on the articular side of the implantation. (Fig. 3, *B*). The implantation gap is calculated at the base of the implantation site. The congruence step-off is measured by comparing the implant height with the radius of best fit using 3 points of the humeral head.

### Biomechanical force measurements: compression test

**Figure 4:**
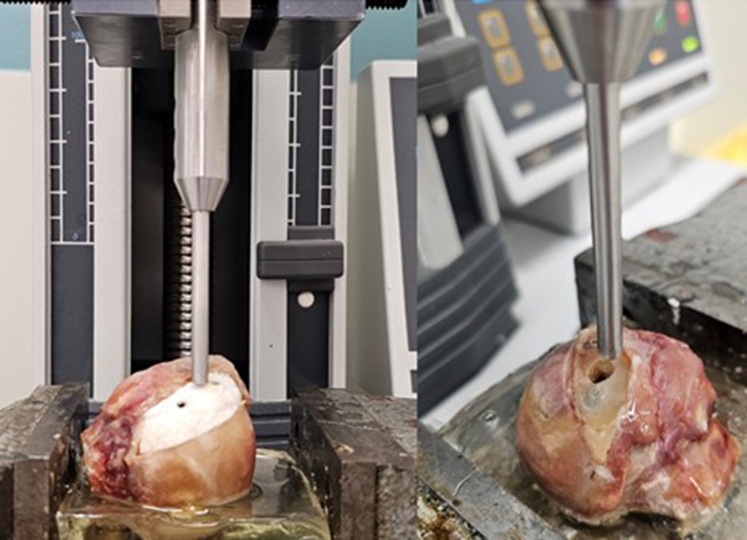
The compression test setup. (Left) Group B: O3D MgP-PCL 3D-printed implant. (Right) Group A: OCA procedure after completion of the compression test. *OCA*, osteochondral allograft; *MgP-PCL*, magnesium phosphate polycaprolactone; *O3D*, osteoregenerative 3D-printed implant.

Finally, an allograft and scaffold biomechanical resilience test was performed using a 5 mm indentation rod (Fig. 4). Each implanted humeral head was placed back into the compression setup with their indentation rod placed in between the 2 fixation screws, after which the compression test was performed at a speed of 2.0 mm/s with a preload of 5 N until a depth of 15 mm was reached. The peak force for each specimen was extracted.

### Breakout mechanism

Micro-CT and confocal microscopy were performed to qualitatively assess whether different breakout mechanisms could be identified between the 2 reconstruction groups. Micro-CT was performed using a Quantum FX Perkin Elmer (μCT, Quantum FX; PerkinElmer, Springfield, IL, USA). The implants were scanned at 90 kV tube voltage, 180 mA tube current, 30 μm resolution, and 3 min scan time. Briefly, the 3D scans of the implants were adjusted using ImageJ software (National Institute of Health, Bethesda, MD, USA) based on the Bernsen thresholding method. Furthermore, constructs were imaged using a confocal microscope (Leica SP8X Laser Scanning, Leica Microsystems, Wetzlar Germany) and Leica LASX (Leica Microsystems, Wetzlar, Germany) acquisition software.

### Statistics

Variance was first found using the Levene's test. Next the Student *t* test was used to compare the compression force, wedge surface area, joint surface area, and wedge volume of the allograft implant and the MgP-PCl 3D-printed Implant. SPSS statistical software (IBM Corp., Armonk, NY, USA) was used for all statistical analysis, with the *P* level set at .05.

## Results

### Morphological measurements

**Figure 5:**
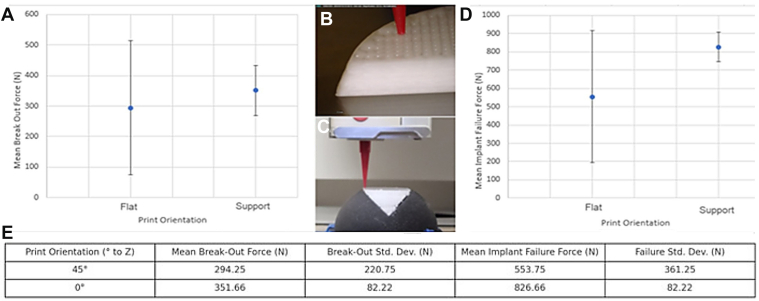
(**A**) The peak breakout force compared between 2 print orientations: Z-direction printing at 45° and 0°. (**B**) Printing orientation at 45° during fabrication with MgP-PCL. (**C**) Printing orientation at 0° using the same ceramic-polymer ink with a nonpersonalized nylon support. (**D**) Mean implant failure force per Z-direction (flat print corresponds to 45° printing; support print corresponds to 0° printing). (**E**) Overview of mean breakout force and mean implant failure force compared across printing orientations with and without support.

Prior to 3D printing of the Hill-Sachs defects, the first step was to see what form of 3D printing internal orientation yielded the highest compression force. Seven flat prints were compared to 7 prints using a support structure, as illustrated in Figure 5.

**Figure 6:**
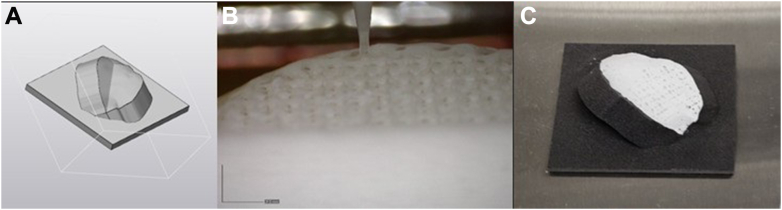
Extrusion-based 3D printing of the 3D-printed MgP-PCL bone regenerative implant using the CT-scan–personalized nylon support. (**A**) Mimics design of the humeral head placed on the custom support design. (**B**) Printing of the 30% MgP-PCL personalized implant. (**C**) The custom-made osteoregenerative implant used in the O3D print group, tailored to the cadaveric humerus CT scan. *CT*, computed tomography; *MgP-PCL*, magnesium phosphate polycaprolactone.

Due to the higher mean break out force as well as higher mean implant failure force the print orientation of 0° in relation to the Z-direction was used for the final 3D-printed MgP-PCL Hill-Sachs implants. As a result, all further prints were designed to be personalized to fit and adapted to the humeri CT scans, and printed as illustrated in Figure 6.

### Specimen characteristics after randomization

The 14 native humeral heads were randomized prior to the experiments. The 2 groups were evaluated before implantation based on radius, surface area, and volume. No significant differences were observed between the groups following randomization, as shown in Table I.

**Table I:** Measurement characteristics of the humeri predefect

	Native humeri n = 14	Group A/OCA n = 7	Group B/O3D n = 7
Radius (mm ± SD)	24.0 ± 2.4	23.3 ± 3.1	25.1 ± 1.7
Surface area (cm^2^ ± SD)	0.73 ± 0.18	0.64 ± 0.17	0.79 ± 0.11
Volume (cm^3^ ± SD)	6.0 ± 2.0	5.0 ± 2.0	6.7 ± 2.0

*SD*, standard deviation; *OCA*, osteochondral allograft; *O3D*, osteoregenerative 3D-printed implant.

### Biomechanical measurements

#### Simulated Hill-Sachs fracture

**Figure 7:**
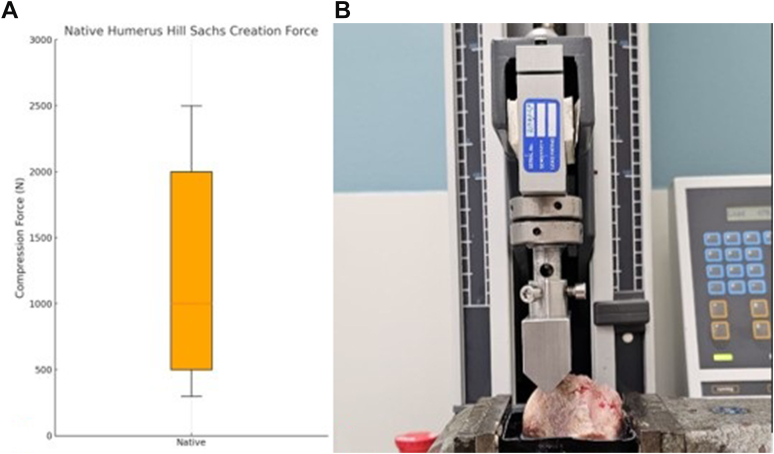
(**A**) Box-and-whisker plot representing the compression force (N) applied to all 14 native humeri to create the large HSL. (**B**) The LR5K Universal Testing Machine, equipped with an XLC 5 kN load cell (Lloyd Instruments Inc., Hampshire, UK), was utilized to generate the Hill-Sachs defect. A custom-fabricated metal triangular sawbone model (simulating the glenoid) was rigidly secured within the biomechanical compression arm, orienting the glenoid model at a 90° impaction angle relative to the epoxy-embedded humeral head, which was fixed in a vice at a position 1 cm medial to the bare area. *HSL*, Hill-Sachs lesion.

The average peak force to create an HSL using the glenoid-mimicking indentation block was 1,120 ± 705 N, as illustrated in Figure 7.

#### Hill-Sachs postoperative implant measurements

The size accuracy of the implants after implantation was evaluated using CT-scan and postprocessing and are displayed in Figure 3. Table II presents the comparison of surface area, joint surface area, and volume between the 2 implantation groups. The surface area, joint surface area, and volume of the wedges were not statistically significantly different (at *P* = .80) between group A and group B.

**Table II:** Accuracy of surface area, joint surface area, and volume of implanted wedges comparison of group A to group B

	Group A/OCA	Group B/O3D
Surface area wedge (mm^2^ ± SD)	1,460 ± 250	1,523 ± 323
Joint surface area (mm^2^ ± SD)	728 ± 125	820 ± 214
Volume wedge (mm^3^ ± SD)	2,915 ± 756	3,062 ± 1,402

*SD*, standard deviation; *OCA*, osteochondral allograft; *O3D*, osteoregenerative 3D-printed implant.

### Surgical placement accuracy

The accuracy of surgical placement was evaluated by measuring the articular step-off, implantation gap, and congruence step-off on postimplantation imaging. (Table III). The articular step-off, implantation gap, and congruence step-off were measured as illustrated in Figure 4. An optimal congruence, articular step-off, and implantation gap is established when <1 mm has been established.

**Table III:** Accuracy of the implantation method of implanted wedges

	Group A/OCA	Group B/O3D
Articular step-off (mm ± SD)	0.73 ± 0.34	0.63 ± 0.41
Implantation gap (mm ± SD)	2.21 ± 0.95	1.60 ± 0.69
Congruence step-off (mm ± SD)	3.17 ± 1.43∗	1.26 ± 0.29∗

*SD*, standard deviation; *OCA*, osteochondral allograft; *O3D*, osteoregenerative 3D-printed implant.

∗ Statistically significant difference at *P* = .044.

The articular step-off, implantation gap, and congruence step-off of the wedges were not statistically significantly different (at *P* = .87) between group A and group B. The articular step-off was found to be accurate (<1 mm) for both implantation groups. The implantation gap was found to be acceptably accurately (1-3 mm) placed for both implantation groups, without statistical significance between both groups (*P* = .54). The congruence step-off was found to be statistically different (*P* = .044) in favor of the O3D Implant group over the OAC implant group. The distance between both screws was measured as 9.8 ± 0.3 mm and was not statistically different between both groups (*P* = .43).

#### Breakout compression test

**Figure 8:**
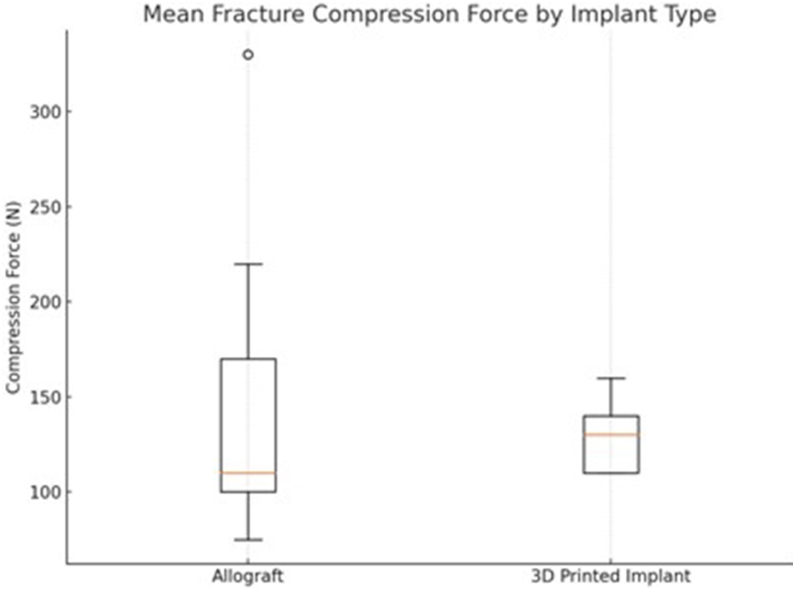
Box-and-whisker plot illustrating the testing conditions of group A (OCA) and group B (O3D), compared by breakout force (N). The indentation had a diameter of 0.5 cm, with a vertically applied compression force. *OCA*, osteochondral allograft; *O3D*, osteoregenerative 3D-printed implant.

The mean biomechanical resilience compression test 144 ± 37N exhibited by the 3D-printed MgP-PCL implant was not statistically significantly different (*P* = .80) in comparison to the femur head osteochondral allograft implant. (Fig. 8). However, the variation in outcomes (standard deviation) was lower.

#### Breakout mechanism

**Figure 9:**
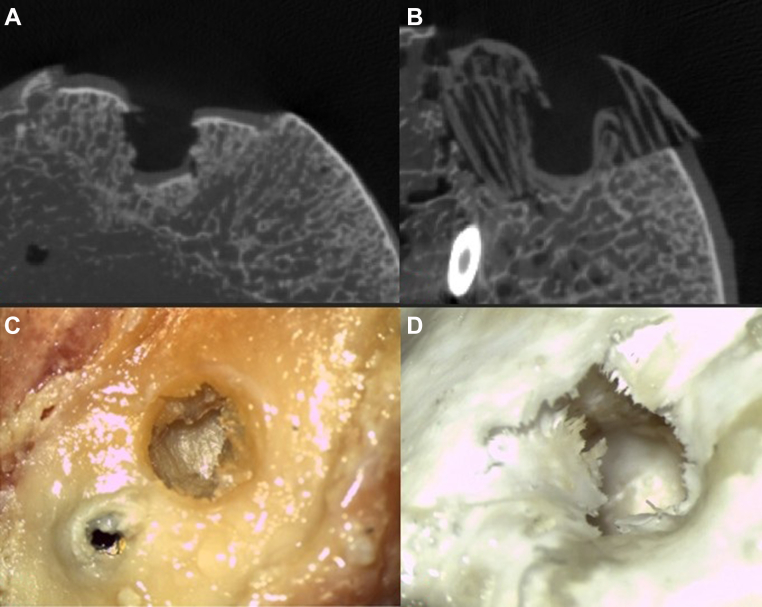
Microscopy images after breakout testing: (**A**) group A — femur OCA; (**B**) group B — O3D MgP-PCL 3D-printed osteoregenerative implant. Note: the cortical screw fixation displacement after breakout in both groups. Micro-CT scans visualizing the breakout results: (**C**) group A — OCA/femur OCA; (**D**) group B — O3D 3D-printed MgP-PCL implant. The analysis revealed differing failure mechanisms: cortical compression in OCA vs. layer deformation in O3D. *OCA*, osteochondral allograft; *CT*, computed tomography; *MgP-PCL*, magnesium phosphate polycaprolactone; *O3D*, osteoregenerative 3D-printed implant.

Micro-CT imaging clearly highlighted the structural differences between the fiber architecture of the 3D-printed implant and the trabecular composition of the femoral allograft bone. Qualitative assessment of the postindentation micro-CT data, in combination with confocal microscopy, suggested that the fibers of the 3D-printed implant were more susceptible to tearing, whereas the trabecular structure of the allograft bone appeared more prone to buckling, as illustrated in Figure 9.

## Discussion

This study compared 2 reconstruction materials—allograft and 3D-printed MgP-PCL 30%—for the repair of critical sized HSLs in cadaveric humeral heads, focusing on both surgical congruency and mechanical performance. The surface area and volume of the implants for both groups were found to be equivalent to a “large” HSL, hence confirming a critical defect requiring surgical intervention.^5^ Postreconstruction CT analysis confirmed accurate implant placement in both groups, with articular step-off consistently below 1 mm and no significant difference in implantation gap. However, congruence step-off was significantly smaller in the 3D-printed group compared to the allograft group. For reference, Ganokoroj et al^5^ reported an accurate postaugmentation congruence of 1.95 ± 0.23, which was successfully achieved in the 3D-printed implantation group. The 3D-printed scaffold group also demonstrated a slightly higher minimum breakout force (103 N) than the allograft group (75 N), but was not significantly different. Detailed assessment of the failure mechanism provided some insight in how both materials react different under biomechanical stress.

Even small incongruences in the humeral head joint surface can disturb load distribution and joint mechanics, potentially accelerating cartilage wear and joint degeneration and despite being small, the congruence step-off was significantly smaller in the 3D-printed group compared to the allograft group. One of the reasons for the difference is that the allograft was shaped until a good fit was found during surgery, while the 3D print was printed in the size that was measured on CT, plus an additional millimeter for a better press-fit. Incorporation of the 3D print may have affected its accuracy. In future applications, a hybrid approach—using preoperative 3D printing with materials that allow limited intraoperative adaptation—may optimize implant fit and congruency.

The 3D-printed scaffold group also demonstrated a higher minimum breakout force (103 N) than the allograft group (75 N). These forces approach or exceed thresholds reported for early functional loading of the shoulder in overhead work, such as 85°-140 N described by Rempel et al.^19^ Two key factors must be considered: first, the 3D-printed MgP-PCL implant serves as a scaffold for bone ingrowth and is not expected to bear the full mechanical load immediately after implantation; second, the allograft group may have been affected by donor-specific variations in bone quality, including undiagnosed osteoporosis. Despite these limitations, the low standard deviation observed in the 3D-printed group suggests a more predictable biomechanical performance.

Postindentation micro-CT and confocal microscopy revealed distinct failure mechanisms between the groups. The 3D-printed implants showed fiber tearing, while the allografts demonstrated trabecular buckling—findings that correspond to the differing material properties of each construct. Both groups exhibited deformation around the screw and indentation zones, as expected when compressing composite materials against dense titanium screws. These insights reinforce the importance of understanding material behavior under axial loading in reconstructive procedures.

A key advantage of the 3D-printed MgP-PCL scaffold is the elimination of risks associated with allograft use, including disease transmission, immune rejection, and variable resorption due to limited cellular activity. The MgP-PCL scaffold's customizable degradation profile offers potential for controlled resorption and structural stability during bone regeneration. While novel strategies such as cartilage-mimicking outer layers and stem-cell–seeded scaffolds are under development, they are not yet widely approved or biomechanically validated.^14^^,^^22^ The current choice of an approved scaffold material allows for clinically relevant application with a clear safety profile.

Several limitations of this study should be acknowledged. First, the average age of cadaveric donors (82.5 yr) likely affected baseline bone quality, reducing the generalizability of findings to younger trauma patients with HSLs.

Second, although the HSLs creation method using the uniaxial biomechanical loading machine ensured standardization, it differs from real-world HSL formation, which typically involves translational forces during dislocation. However, a similar method was published prior by Itoi et al^12^ and was therefore repeated in our study design. In addition, biomechanical studies are inherently limited in sample size, as noted by our small sample size of 7 per study group.

Third, this study focused exclusively on reconstructive accuracy and implant resilience, not on global shoulder stability, which also depends on soft tissue structures such as the rotator cuff and capsuloligamentous complex. This approach was chosen to isolate the mechanical properties of the grafts and scaffolds without confounding variables. Fourth, the process of a visual inspection and manual reshaping have been taken into account, just as one performed in the modern day clinical practice with OCA. This process could be improved by making a detailed allograft sizing and a donor matching protocol.

Finally, while the regenerative potential of the MgP-PCL scaffold has been demonstrated in vivo,^7^ such biological effects could not be assessed in a cadaveric setting. These findings nonetheless support the potential for clinical translation, which should be further validated through in vivo studies evaluating osteointegration, immune response, and long-term joint function.

## Conclusion

Both allograft implants and 3D-printed MgP-PCL implants demonstrate a comparable potential in restoring the surface area of the shoulder after a large HSL. The 3D-printed MgP-PCL implants demonstrated significantly superior congruency when compared to the OCA implant, indicating a potential advantage in joint alignment. Furthermore, the breakout force for the allograft procedure was comparable to the MgP-PCL implant, suggesting comparable mechanical stability.

Our successful morphological measurements in combination with previously successful in vivo results demonstrating favorable host response and osseointegration, warrants this approach for further investigation. Future research should focus on evaluating long-term mechanical performance under physiologic loading and joint motion.

Translational efforts should prioritize scaling the implant for human anatomical variation, optimizing surgical techniques, and aligning with regulatory requirements. Key considerations include biocompatibility, degradation kinetics, and functional performance in load-bearing environments to support eventual clinical application.

## Acknowledgments

The authors acknowledge the technical help of Eva F.G.J. Stronkman, Mattie van Rijen, and Joost H. van Duijn at the Department of Orthopaedics, University Medical Center Utrecht, Utrecht, The Netherlands.

## Disclaimers

Funding: Stichting Vrienden van her UMC Utrecht. The funder had no role in data collection, data analysis, or the preparation of or editing of the manuscript. This work was supported by the PRosPERoS-II project, funded by the Interreg VA Flanders-the Netherlands program, CCI grantno. 2021TC16RFCB041.

Conflicts of interest: Bart van der Wal holds minority shares in Replasia B.V. Amotio B.V., Preseurgeo Uplanner B.V., and Preimure B.V. Michel van den Bekerom indicated fellowship support from Smith & Nephew PLC. Arthur van Noort indicated being a key opinion leader of Enovis Corp (Previously LIMACorporate) and involvement in fellowship support from Enovis Corp. The other authors, their immediate families, and any research foundation with which they are affiliated have not received any financial payments or other benefits from any commercial entity related to the subject of this article.
